# Supplementary material for: Validation of the HFA-ICOS Score for Carfilzomib-Induced Cardiotoxicity in Multiple Myeloma: A Real-Life Perspective Study
Source: Cancers (Basel). 2025 Jul 15;17(14):2353. doi: 10.3390/cancers17142353 (PMC12293333; doi:10.3390/cancers17142353)
Supplement: Supplementary file 1 [file cancers-17-02353-s001.zip › cancers-3645449-supplementary.pdf]

Supplementary Materials

Table S1. Carfilzomib-based regimens of the population.

**Protocol**	**Chemotherapeutic Agents**	**Administration days<br>(Cycles of 28 days)**	**Patients<br>N 169 (%)**
KD	- Carfilzomib 56mg/m_2<br>(20mg/m_2 D1 and D2 of C1)<br>- Dexamethasone 20 mg	- D1, 2, 8, 9, 15, 16<br><br>- D1, 2, 8, 9, 15, 16, 22, 23	60 (35.5)
KRD	- Carfilzomib 27 mg/ m_2<br>(20mg/m_2 D1 and D2 of C1)<br>- Dexamethasone 40 mg<br>- Lenalidomide 25 mg	- D1, 2, 8, 9, 15, 16<br>(from C13: 1, 2, 15, 16)<br>- D1, 8, 15, 22<br>- From D1 to D21	67 (39.6)
FORTE<br>*Phase III study in newly diagnosed MM*	*Study arm 1 (ASCT after 4 cycles):*<br>- Carfilzomib 36 mg/m_2<br>(20mg D1 and D2 of C1)<br>- Dexamethasone 40 mg<br>- Lenalidomide 25 mg	 <br>- D1, 2, 8, 9, 15, 16<br><br>- D1, 2, 8, 9, 15, 16, 22, 23<br>- From D1 to D21	6 (3.5)
	Study arm 2:<br>- Carfilzomib 36 mg/m_2<br>(20mg D1 and D2 of C1)<br>- Dexamethasone 40 mg<br>- Lenalidomide 25 mg	 - D1, 2, 8, 9, 15, 16<br><br>- D1, 2, 8, 9, 15, 16, 22, 23<br>- From D1 to D21	
	Study arm 3 (ASCT after 4 cycles):<br>- Carfilzomib 36 mg/m_2<br>(20mg D1 and D2 of C1)<br>- Dexamethasone 20 mg<br>- Cyclophosphamide 300 mg/m2	 <br>- D1, 2, 8, 9, 15, 16<br><br>- D1, 2, 8, 9, 15, 16, 22, 23<br>- D1, 8,15	
EMN07<br>*Phase I/II study in RRMM*	- Carfilzomib 27/36/45/56 mg/m2<br>(level -1/0/+1/+2) (20mg/m_2 D1 of C1)<br>- Dexamethasone 20 mg<br>- Pomalidomide 4 mg	- D1, 8, 15<br><br><br><br>- D1, 8, 15, 22<br>- From D1 to 21	13 (7.7)
ARROW<br>*RRMM*<br>*Phase III study in RRMM*	*Study arm A:*<br>- Carfilzomib 70 mg/m_2<br>(20mg/m_2 D1 of C1)<br>- Dexamethasone 40 mg	 - D1, 8, 15<br>- D1, 8, 15 (22 from C1 to C9)	2 (1.2)
	Study arm B:<br>- Carfilzomib 27 mg/m_2<br>(20mg/m_2 D1 and D2 of C1)<br>- Dexamethasone 40 mg	 - D1, 8, 15 (22 from C1 to C9)<br><br>- D1, 2, 8, 9, 15, 16	3 (1.7)
EMN11<br>*Phase I/II study in RRMM*	(ASCT after 4 cycles)<br>- Carfilzomib 36 mg/m_2<br>(20mg/m_2 D1-D2 of C1)<br>- Dexamethasone 20 mg<br>- Pomalidomide 4 mg	 - D1, 2, 8, 9, 15, 16<br><br>- D1, 2, 8, 9, 15, 16<br>- From D1 to D21	3 (1.7)
EMN24<br>*Phase III study in newly diagnosed MM eligible for autologous stem cell transplantation (IsKia trial)*	(ASCT after 4 cycles)<br>KRD induction<br>- Carfilzomib 56 mg/m_2<br>(20mg D1 and D2 of C1)<br>- Lenalidomide 25 mg<br>- Dexamethasone 40 mg	 <br>- D1,8,15<br><br>- From D1 to D21<br>- D1,8,15,22	9 (5.3)

	(ASCT after 4 cycles)<br>Isa- KRD induction<br>-Isatuximab 10 mg/m^2<br>- Carfilzomib 56 mg/m^2<br>(20mg D1 and D2 of C1)<br>- Lenalidomide 25 mg<br>- Dexamethasone 40 mg	 <br>- D1,8,15,22<br>- D1,8,15<br><br>- From D1 to D21<br>- D1,8,15,22	6 (3.5)
